# Task shifting between physicians and nurses in acute care hospitals: cross-sectional study in nine countries

**DOI:** 10.1186/s12960-018-0285-9

**Published:** 2018-05-25

**Authors:** Claudia B. Maier, Julia Köppen, Reinhard Busse, MUNROS team, Christine Bond, Robert Elliott, Hanne Bruhn, Debbie Mclaggan, Daryll Archibald, Mandy Ryan, Diane Skatun, Sebastian Heidenreich, Frantisek Vlcek, Marie Zvonickova, Daniel Hodyc, Hana Svobodová, Matthew Sutton, Jonathan Gibson, Anne McBride, James McDonald, Steve Birch, Britta Zander, Juliane Stahl, Silvia Coretti, Paola Codella, Matteo Ruggeri, Job van Exel, Antoinette de Bont, Marianne Luyendjk, Iris Wallenburg, Apostolos Tsiachristas, Maarten Janssen, Mathijs Kelder, Maureen Rutten-van Molken, Jan Erik Askildsen, Muhammad Kamrul Islam, Jon Opsahl, Linda Ostergren, Nina Berven, Kjell Haug, Bjarte Folkestad, Kari Ludvigsen, Bodil Ravneberg, Alicja Sobczak, Grazyna Dykowska, Malgorzata Winter, Sabina Ostrowska, Michal Mijal, Sinem Erincç, Seda Basihos, Meryem Dogan, Güldem Ökem

**Affiliations:** 1 Department of Healthcare Management, Technische Universität Berlin, H 80, Straße des 17. Juni 135, 10623 Berlin, Germany; 2 Center for Health Outcomes and Policy Research, University of Pennsylvania, School of Nursing, Claire Fagin Hall, 418 Curie Blvd., Philadelphia, PA 19104 United States of America

**Keywords:** Health professionals, Physicians, Nurses, Task shifting, Advanced practice nursing, Scope-of-practice, Hospitals, Clinical practice, Breast cancer, Acute myocardial infarction, Gesundheitsfachkräfte, Ärzte, Pflegekräfte, Übertragung ärztlicher Tätigkeiten auf Pflegekräfte, Delegation, Substitution, Pflegeexperte APN, Advanced Practice Nurse/Nurse Practitioner, Offizielle Aufgabenbereiche, Krankenhaus, Klinische Tätigkeit, Brustkrebs, Akuter Myokardinfarkt, Herzinfarkt

## Abstract

**Background:**

Countries vary in the extent to which reforms have been implemented expanding nurses’ Scopes-of-Practice (SoP). There is limited cross-country research if and how reforms affect clinical practice, particularly in hospitals. This study analyses health professionals’ perceptions of role change and of task shifting between the medical and nursing professions in nine European countries.

**Methods:**

Cross-sectional design with surveys completed by 1716 health professionals treating patients with breast cancer (BC) and acute myocardial infarction (AMI) in 161 hospitals across nine countries. Descriptive and bivariate analysis on self-reported staff role changes and levels of independence (with/without physician oversight) by two country groups, with major SoP reforms implemented between 2010 and 2015 (Netherlands, England, Scotland) and without (Czech Republic, Germany, Italy, Norway, Poland, Turkey). Participation in ‘medical tasks’ was identified using two methods, a data-driven and a conceptual approach. Individual task-related analyses were performed for the medical and nursing professions, and Advanced Practice Nurses/Specialist Nurses (APN/SN).

**Results:**

Health professionals from the Netherlands, England and Scotland more frequently reported changes to staff roles over this time period vs. the other six countries (BC 74.0% vs. 38.7%, *p* < .001; AMI 61.7% vs. 37.3%, *p* < .001), and higher independence in new roles (BC 58.6% vs. 24.0%, *p* < .001; AMI 48.9% vs. 29.2%, *p* < .001). A higher proportion of nurses and APN/SN from these three countries reported to undertake tasks related to BC diagnosis, therapy, prescribing of medicines and information to patients compared to the six countries. Similar cross-country differences existed for AMI on prescribing medications and follow-up care. Tasks related to diagnosis and therapy, however, remained largely within the medical profession’s domain. Most tasks were reported to be performed by both professions rather than carried out by one profession only.

**Conclusions:**

Higher levels of changes to staff roles and task shifting were reported in the Netherlands, England and Scotland, suggesting that professional boundaries have shifted, for instance on chemotherapy or prescribing medicines. For most tasks, however, a partial instead of full task shifting is practice.

## Abstrakt

**Hintergrund:**

Viele Länder in Europa variieren in dem Ausmaß, in dem Reformen zur Aufgabenerweiterung von Pflegekräften durchgeführt wurden. Länderübergreifende Forschung ist bisher unzureichend, ob und wie Reformen die klinische Praxis in Krankenhäusern beeinflussen. Diese Studie analysiert die Rollenveränderungen und die Übertragung von Aufgaben zwischen Ärzten und Pflegekräften in neun europäischen Ländern.

**Methodik:**

Querschnittstudie, basierend auf einer Fragebogenerhebung von 1716 Gesundheitsfachkräften in 161 Krankenhäusern in neun Ländern, die Patienten mit Brustkrebs (BK) und akutem Myokardinfarkt (AMI) behandeln. Deskriptive und bivariate Analysen zur Rollenveränderung der Mitarbeiter, Autonomie (mit/ohne ärztlicher Aufsicht) wurden anhand zweier Ländergruppen durchgeführt: mit Reformen bzgl. Erweiterung der offiziellen Aufgabenbereiche (*Scope-of-Practice*), die zwischen 2010 und 2015 implementiert wurden (Niederlande, England, Schottland) und Länder ohne Reformen (Deutschland, Italien, Norwegen, Polen, Tschechien, Türkei). Darüber hinaus wurden 'medizinische' Tätigkeiten identifiziert, mit einem datenbasierten und einem konzeptionellen Ansatz. Die Analysen wurden für Ärzte, Pflegekräfte sowie für Advanced Practice Nurses/andere Spezialisierungen (APN/SN) separat durchgeführt.

**Ergebnisse:**

Gesundheitsfachkräfte aus den Niederlanden, England und Schottland berichteten häufiger von Rollenveränderungen (BK 74.0% vs. 38.7%, *p* < .001; AMI 61.7% vs. 37.3%, *p* < .001); sowie höherer Autonomie in den neuen Rollen (BK 58.6% vs. 24.0%, *p* < .001; AMI 48.9% vs. 29.2%, *p* < .001), verglichen mit den anderen sechs Ländern. Pflegekräfte und insbesondere APN/SN aus den drei Ländern berichteten häufiger, dass sie Aufgaben im Zusammenhang mit der Diagnosestellung von Brustkrebs, Therapie, Verschreibung von Medikamenten durchführen und Informationen an Patienten weitergeben. Ähnliche Unterschiede gab es für AMI in Bezug auf die Verschreibung von Medikamenten und Nachsorge, aber nicht im Zusammenhang mit der Diagnosestellung und Therapie, die weitestgehend im Tätigkeitsbereich der Ärzte verblieb. Die meisten Aufgaben wurden von beiden Berufsgruppen anstatt von einer durchgeführt.

**Schlussfolgerungen:**

In den Niederlanden, England und Schottland wurden vermehrt Rollenveränderungen und die Aufgabenübertragung von Ärzten auf Pflegekräfte berichtet, was darauf hindeutet, dass sich berufliche Zuständigkeitsbereiche verlagert haben, beispielsweise in Bezug auf die Chemotherapie oder das Verschreiben von Medikamenten. Für die meisten Tätigkeiten findet in der Praxis eine teilweise und keine vollständige Aufgabenverlagerung statt.

## Background

The roles and responsibilities of health professionals have undergone changes in many countries over the past decade. Population ageing combined with increasing rates of chronic conditions have increased demand for healthcare services and put pressure on health systems to ensure high-quality, coordinated care [1, 2]. Yet, shortages or geographical maldistribution of health professionals are common and workloads high. At the same time, there is a trend among the medical and nursing professions to undergo further specialisation and training [3–5]. In Europe, the Bologna process has instigated educational reforms for nurses, with higher educational institutions increasingly offering postgraduate programmes expanding nurses’ skills and competencies [6].

Against this backdrop, many countries have expanded the roles for nurses. Examples include Advanced Practice Nurses/Nurse Practitioners (APN/NP) with usually a Master’s level degree or Specialist Nurses (SN) with various educational backgrounds and titles [4, 7]. Nurse role expansion can take two forms: first, task shifting, whereby nurses take up tasks from the medical profession, for instance under physician oversight (delegation) or independently (substitution) [8–11]. Second, task supplementation is defined as nurses complementing existing roles, such as eHealth monitoring or coordination [11, 12]. This study focuses on the former model.

A 2015 study found large variations in nurses’ official Scopes-of-Practices (SoP) across 39 countries. From within Europe, the following countries were identified as having implemented extensive task shifting in primary care: Finland, Ireland, Netherlands, England, Northern Ireland, Scotland and Wales [13]. Other international studies have either focused on APN education and regulation [14–16]; or on primary care, e.g. in Australia, Canada, the Netherlands, the United Kingdom (UK) or the United States of America (USA) [17–20]. A review found that NP are able to perform between 67% to 93% of all primary care services, based on few high-quality studies [21].

There is limited evidence on the exact clinical activities which APN/NP perform in hospitals. In the European Union (EU) Framework Programme 7 (FP7) funded MUNROS project,^1^ it was found that the roles of health professionals have changed in all nine countries studied, but most changes occurred in the Netherlands, England and Scotland [22]. In the Netherlands, a case study in five hospitals analysed prescribing among Nurse Specialists^2^ with Master’s level degree NP [23]. This group of nurses varied within and between hospitals in their right to prescribe medications, partly due to differences in individual hospital-oversight measures in place. In England, a case study evaluated the impact of introducing NP^3^ in one hospital [24] and found NP to undertake tasks traditionally performed by junior doctors, with positive impacts on patient experience and outcomes.

To date, no international study has analysed the detailed tasks of nurses, APN/NP and/or SN and compared them to physicians in hospitals. Against this backdrop, the purpose of this paper was to assess if countries with reforms related to expanding the SoP were associated with reported staff role changes and task shifting between the medical and nursing professions, for BC and AMI. The study is of relevance from an international and European perspective. In the EU’s single market where physicians and nurses are free to move and work in any other participating country, an assessment of the routine tasks of nurses and physicians will allow for enhanced cross-country comparability in hospital settings.

## Methods

The objectives of this paper were twofold: first, to analyse health professionals’ perception of staff role changes and their independence in new roles (with/without physician oversight), differentiating between countries that had implemented major vs. no/limited reforms to nurses’ SoP. Second, to analyse task shifting in hospitals at the individual task levels, undertaken by physicians, nurses and APN/SN, using a cross-country, cross-sectional study design.

The study was part of the EU-MUNROS research in nine countries: Czech Republic, England, Germany, Italy, Netherlands, Norway, Poland, Scotland and Turkey. England and Scotland were surveyed separately due to the devolved governance structure of the UK National Health Services. Countries were selected for their variations in their health workforce and health systems [22, 25].

### Identification of country reforms

For the nine countries, two country groups were identified depending on whether major policy reforms, expanding the SoP for nurses, had been implemented between 2010 and 2015 [13]. The first group, hereafter referred to as ‘skill-mix innovator’ countries, included the Netherlands, England and Scotland (Table 1). In the Netherlands, a 2011 law entering into force in 2012 expanded the SoP for Nurse Specialists (and Physician Assistants) by 11 tasks which were previously reserved to the medical profession only. These tasks included prescribing medications, performing surgeries, and giving injections, among others [26]. Moreover, since 2014, three groups of nurses (diabetes, lung and oncology nurses) have been allowed to prescribe medications following protocols and after the initial diagnosis by a physician. In England, Northern Ireland, Scotland and Wales, independent nurse prescribing was introduced in 2006. The law allowed nurses (and pharmacists) who had successfully completed an independent prescribing course to prescribe any licenced medicine (other than some controlled drugs) for any medical condition within their clinical competence [27]. In 2012, the restriction on controlled drugs was removed putting nurses on a par with doctors in relation to prescribing capabilities.

**Table 1:** Policy reforms by country: major SoP reforms expanding nurses’ clinical practice implemented; 2010–2015

	Expanded SoP for APN/nurses	No/limited expansions to SoP
Netherlands	• 2011 Law expanded the SoP for Nurse Specialists • 2014 Law on nurse prescribing for three nurse specialisations	
England (UK)	• Prescribing rights expanded for independent prescribers (2012)	
Scotland (UK)	• Prescribing rights expanded for independent prescribers (2012)	
Czech Republic		• Limited nurse role developments, no changes to SoP
Germany		• Limited nurse role developments • 2011: Directive to authorise pilot projects to test task shifting
Italy		• Limited developments, no changes to SoP
Norway		• Limited APN/nurse role developments, no changes to SoP
Poland		• Limited developments, except for a law on nurse prescribing which entered into force in 2016^a^
Turkey		• No developments, no changes to SoP

Source: [13, 26, 27, 35, 36]

*SoP* Scope-of-Practice, *APN* Advanced Practice Nurse, *UK* United Kingdom

^a^Poland was categorised as “limited/no reforms”, due to the fact that the law entered into force in 2016 (outside the 2010–2015 time period covered)

The second group of countries—hereafter referred to as ‘traditional skill-mix’ countries—with no or limited SoP reforms included the remaining six countries, relying on a traditional SoP for nurses, although in some countries pilot projects existed.

### Health professional survey

As part of the MUNROS research, a survey was conducted among health professionals, healthcare managers and patients, focusing on three prevalent conditions: BC, AMI and type 2 diabetes. Data collection took place from 2015 to 2016. Detailed accounts of the study design are available elsewhere [25]. Sampling followed a non-representative, purposive sampling strategy involving hospitals and their associated primary care sites. For this analysis, a sub-set of health professionals working in hospitals were included, if actively involved in the hospital-based care of patients with either BC or AMI. Type 2 diabetes was excluded as routine management is predominantly primary care based. The sample comprised 1716 health professionals, including 802 health professionals working on BC (*N* = 76 hospitals) and 914 on AMI (*N* = 85 hospitals). Sub-groups included 231 physicians and 375 professional nurses (of whom 97 were specialised as APN/SN) for BC, and 200 physicians and 550 nurses (132 APN/SN) for AMI.

### Survey instrument

The survey instrument included questions on the specific tasks undertaken along the care pathway by each of the health professionals, to be completed if personally undertaken as part of normal duties. The care pathway was structured according to the various stages, namely BC diagnosis, surgery and other therapy, follow-up/managing complications and palliative care. For AMI, it included diagnosis and assessment, surgery and other therapy, managing complications, rehabilitation and post-discharge/follow-up care. There were 92 tasks on BC and 51 tasks on AMI. The tasks were identified by clinical specialists as typically performed by health professionals working on the pathways [25]. Other sections of the survey included questions about professionals’ qualifications, perceptions of role change over the last 5 years, and whether or not new roles were delivered independently, with or without physician supervision. The survey instrument was developed in English and translated into seven languages, and verified by back translation [28, 29]. Following a pilot study, minor changes were made and the questionnaire was distributed in 2015/2016. Ethical and other relevant approvals were obtained in all countries. Data were first entered into standardised excel spread sheets, cleaned and validity checks undertaken by individual country teams following the study protocol, and later imported into one consolidated database and checked again for plausibility and validity [25]. The process of defining health professions into professional groups (physicians, nurses, APN/SN) was based on two elements, reported job title and qualification. Qualification included questions about basic qualification (e.g. medical or nursing degree), higher degree and other professional qualifications (e.g. specialisations). Two researchers in each country categorised respondents according to a list of 28 pre-defined professions, including minimum level qualifications. Where possible, definitions and minimum qualifications were based on the European Commission’s Professional Qualifications Directive [30, 31]. Nurses with registered nurses-equivalent education were defined as ‘nurses’, whereas nurses with Master’s degree or other specialisations were defined as APN and SN. However, due to the small sample sizes in the two groups, we merged APN and SN into one group (referred to as APN/SN) for the purpose of this paper.

### Data analysis

Since this study focused on task shifting between the medical and nursing profession, we identified ‘medical’ tasks from the full lists. Two approaches were used: first an empirical, data-driven, and second, a conceptual approach. The empirical method was based on defining tasks as ‘medical’ if reported by the majority of physicians surveyed. Hence, inclusion criteria were all tasks reported by at least 50% of the physicians in at least 50% of the countries. Since the remit of the medical professions’ activities may vary across countries, we used the cut-off of 50% across countries. In addition, we followed the International Council of Nurses (ICN) [7], suggesting APN/NP practice as including the following tasks: diagnoses, ordering tests, treatment/therapy decisions, prescribing, first point of contact, panel of patients, and referrals. Hence, we included tasks from the task lists if directly related to these clinical activities. Exclusion criteria were non-medical tasks, such as making beds, assisting patients with daily activities, physiotherapy or administrative tasks.

Data analyses included descriptive and bivariate analyses, using STATA 14©. Descriptive analyses comprised the total and relative numbers of all health professionals reporting staff role changes, and the reported degree of independence in task shifting (with/without physician supervision). For the individual tasks, data analysis covered physicians, all professional nurses and the sub-group of APN/SNs, by country group (innovator vs. traditional). For each of the outcome variables, associations were tested at the bivariate levels, using chi-squared and Fisher’s exact (two-sided), depending on the sample sizes. Standard levels of statistical significance were used (*p* < .05).

## Results

### Changes to professional roles

Large cross-country differences existed in health professionals’ perception of staff role change. For BC, 74.0% of health professionals from the innovator group of countries (Netherlands, England, Scotland) reported that there had been changes to staff roles over the past 5 years compared to 38.7% from the traditional countries (*p* < .001) (Table 2). Similar results existed for health professionals working on AMI (61.7% vs. 37.3%, *p* < .001). By individual country, reported changes to staff roles were highest in the Netherlands for BC (78.7%) and Scotland for AMI (65.9%) and lowest in Turkey (BC 23.2%, AMI 21.9%).

**Table 2:** Health professionals’ perceived changes to staff roles (2010/2011–2015/2016) and independence in task substitution (without physician supervision) by country and Scope-of-Practice

	Breast cancer		Acute myocardial infarction	
	Changes to staff roles over past 5 years^a^	Task substitution without physician supervision^b^	Changes to staff roles over past 5 years^a^	Task substitution without physician supervision^b^
	% HP	% HP	% HP	% HP
Countries with SoP expansion [1]	74.0***	58.6***	61.7***	48.9***
Countries with no/limited SoP expansion [2]	38.7***	24.0***	37.3***	29.2***
*By country*				
Netherlands	78.7°	51.3°	65.3	38.2
England (UK)	72.3	64.2	54.4	51.1
Scotland (UK)	73.6	55.3	65.9	53.3
Czech Republic	45.5	18.1	50.7	7.4
Germany	43.2	27.4	29.1	45.7
Italy	30.0°	–°°	64.8	51.4
Norway	38.7°	33.3°	44.2	30.4
Poland	43.5°	17.6°	28.7	36
Turkey	23.2	13.0°	21.9	25.9
*Total average* ^c^	50.8***	41.2***	44.1***	36.9***
*Total N*	*687*	*349*	*790*	*349*

[1] England, Scotland, Netherlands, [2] Czech Republic, Germany, Italy, Norway, Poland, Turkey

*HP* Health Professionals, *SoP* Scope-of-Practice

***p value < .001, ° = *n* < 50 observations, °° = value not shown, because *n* < 10 observations

^a^Wording in questionnaire: “Have staff roles changed on the [breast cancer/AMI, respectively] care pathway within the last 5 years?”

^b^Exact wording: “Tasks formerly done by physicians are now done by new professions or existing professions with new independent roles without supervision”

^c^Pearson chi-squared test comparing country-level mean with total average (all-country mean)

### Independence in the uptake of new roles from physicians

Similarly, there were large differences in the reported levels of independence in taking over tasks from the medical profession. For BC, a statistically significantly higher proportion of health professionals working in the innovator countries compared to traditional countries agreed that tasks that had formerly been carried by physicians are now carried out independently by new professions/roles without physician supervision (58.6% vs. 24.0%). Among health professionals working on AMI, differences between country groups also existed, but were smaller (48.9% vs. 29.2%, *p* < .001) (Table 2).

### Tasks performed by the medical and nursing professions

After application of the two methods to identify medical tasks, for BC, 36 tasks were included (39.1% of all tasks), including 7 via the empirical/data-driven method (50%/50% cut-off), 23 via the ICN definition and 6 via both approaches. For AMI, 29 tasks were included (56.8% of all tasks), of which 10 via the 50%/50% cut-off, 14 via the ICN definition and 5 via both approaches.

### Breast cancer

For BC, for the majority of tasks (*N* =24, 66.7%), differences between physicians practicing in countries with and without expanded SoP were small and non-significant (Table 3). For 12 tasks (33.3%), however, statistically significant differences between the country groups existed, whereby physicians in the Netherlands, England and Scotland reported for 11 out of the 12 tasks that they significantly less often perform these tasks.

**Table 3:** Breast cancer: clinical tasks undertaken by percentage of physicians and nurses, by countries’ SoP, 2015/2016

	% Physicians performing tasks		% Nurses performing tasks (all nurses and APN/SN)			
	Countries with SoP expansion [1]	Countries with no/limited SoP expansion [2]	Countries with SoP expansion [1]	Countries with no/limited SoP expansion [2]	Countries with SoP expansion [1]	Countries with no/limited SoP expansion [2]
Breast cancer	% Physicians, *N* = 91	% Physicians, *N* = 132	% All nurses, *N* = 79	% All nurses, *N* = 242	% APN/SN, *N* = 46	% APN/SN *N* = 43
*Tasks related to diagnosis/physical examination*						
Assessing extent of disease/staging: physical examination^[i,ii]^	53.8	54.5	16.4***	1.6***	21.7**	0.0**
Detecting and treating local recurrence: perform physical examination^[i,ii]^	59.3	63.6	26.5***	2.8***	32.6***	0.0***
Detecting and treating metastatic diseases: perform physical examination^[i,ii]^	51.6	61.3	15.1***	3.3***	17.3**	0.0**
Endocrine/hormonal therapy: perform physical examination^[i]^	40.6	36.3	13.9***	1.6***	15.2	4.6
Biological therapy: perform physical examination^[i]^	10.9***	31.0***	8.8*	3.3*	8.7	9.3
Interpret ultrasound scan^[i]^	23.0***	46.2***	3.8	0.8	6.5	0.0
Clinical interpretation of mammogram^[i]^	42.8	31.0	5.0*	0.8*	8.7	0.0
Clinical interpretation of MRI^[i]^	34.0*	21.9*	2.5	0.8	4.3	0.0
Clinical interpretation of biopsy^[i]^	41.7	30.3	3.8*	0.0*	4.3	0.0
Interpret X-ray^[i]^	27.4	21.9	2.5	0.0	4.3	0.0
Interpret CT scan^[i]^	29.6	21.2	1.2	0.0	2.1	0.0
Interpret bone scan^[i]^	21.9	17.4	1.2	0.0	2.1	0.0
*Tasks related to treatment/therapy*						
Surgical treatment: perform surgical procedures^[i,ii]^	40.6	43.1	2.5	3.3	2.1	2.3
Local recurrence: initiate diagnosing treatment^[i,ii]^	52.7	57.4	21.0***	0.8***	30.4***	0.0***
Metastatic diseases: initiate diagnosing treatment^[i,ii]^	47.2	58.3	15.1***	1.2***	19.5**	0.0**
Chemotherapy: decide on therapy based on lab results and protocols^[i]^	16.4***	44.7***	24.0***	1.6***	30.4***	0.0***
Chemotherapy: revise therapy^[i]^	12.0***	38.6***	22.7**	8.2**	30.4***	2.3***
Endocrine/hormonal therapy: prescribe therapy^[i]^	45.0	37.8	12.6**	4.1**	19.5**	0.0**
Endocrine/hormonal therapy: revise therapy^[i]^	43.9	34.8	13.9***	1.2***	19.5**	0.0**
Biological therapy: prescribe therapy^[i]^	12.0**	31.0**	8.8	5.7	13.0	4.6
Biological therapy: revise therapy^[i]^	12.0**	27.2**	11.3**	1.6**	15.2*	0.0*
Lymphedema: initiate treatment^[i]^	23.0	33.3	24.0*	12.4*	30.4	20.9
Cancer-related fatigue: prescribe therapy^[i]^	7.6***	31.0***	30.3***	7.8***	41.3**	9.3**
*Tasks related to prescribing medications*						
Prescribe medication related to treatment^[i]^	37.3	46.9	10.1**	2.0**	13.0*	0.0*
Adapt medication treatment in the course of therapy^[i]^	30.7	42.4	16.4**	4.9**	21.7**	0.0**
Side effects: prescribe medications^[i]^	27.4**	49.2**	10.1**	2.0**	15.2	2.3
Administer medications^[i]^	5.4***	26.5***	17.7**	35.5**	17.3	32.5
Palliative care: prescribe medications^[i]^	17.5**	37.8**	6.3	4.1	10.8	0.0
Palliative care: administer medications^[i]^	0.0***	13.6***	21.5***	50.4***	21.7	37.3
*Tasks related to providing information to patients*						
Providing information to patient about test results^[ii]^	61.5	50.0	35.2***	2.0***	44.6***	6.0***
Detecting and treating local recurrence: inform patients about results^[ii]^	53.8	54.2	23.5***	3.1***	34.0***	4.0***
Detecting and treating metastatic diseases: inform patient about results^[ii]^	50.5	52.1	16.4***	0.6***	21.2***	0.0***
Providing information to patient about clinical aspects and perspective of treatment^[ii]^	58.2	46.4	38.8***	4.8***	48.9***	10.0***
Surgical treatment: inform patient about surgical treatment^[ii]^	46.1	52.8	30.5***	11.3***	36.1*	14.0*
Endocrine/hormonal therapy: inform patient about therapy^[ii]^	50.5	42.8	32.9***	7.9***	42.5**	18.0**
Information to patient about non-clinical consequences of treatment^[ii]^	42.8	40.7	40.0***	5.5***	53.1***	14.0***

Source: MUNROS 2015/2016. Rationale for inclusion of tasks: ^[i]^ = suggested clinical activities as Advanced Nursing Practice: diagnosis/advanced health assessment, treatment/therapy, prescribing medications, by International Council of Nurses (ICN), ^[ii]^ = common medical tasks, reported by the majority of physicians as being part of normal duties (≥ 50% of physicians in ≥ 50% of countries). [1] England, Scotland, Netherlands, [2] Czech Republic, Germany, Italy, Norway, Poland, Turkey

*SoP* Scope-of-Practice, *APN* Advanced Practice Nurses, *SN* Specialist Nurses

***p value < .001; ** = *p* value < .01, * = *p* value < .05

Among the category of all professional nurses on the contrary, for the majority of tasks (*N* =28, 77.7%), nurses in the innovator compared to the traditional countries were statistically significantly more likely to report that they carried out these 28 tasks, covering each of the four stages of the care pathway. For instance, an up to 30-fold higher proportion of APN/SN in the innovator compared to the traditional countries reported performing physical examinations to assess the extent of disease/staging and to detect and treat local recurrence, prescribing therapy on cancer-related fatigue and deciding on and revising chemotherapy (*p* < .001 each). For chemotherapy and therapy for cancer-related fatigue, the proportion of APN/SN and nurses reporting that they undertook these tasks was higher than among physicians in the innovator countries, suggesting that task shifting between the two professions had occurred. In the traditional countries, deciding on and revising chemotherapy was largely undertaken by physicians (44.7% and 38.6%), while low percentages among all nurses (1.6% and 8.2%) and even less in the sub-group of APN/SN (0.0% vs. 2.3%) reported performing these tasks.

Professional nurses in the innovator countries were significantly more likely to report that they prescribed medications as part of their regular work. Moreover, the proportion of physicians in this country group compared to the traditional countries was lower on prescribing medicines to manage side effects (27.4% in the innovator countries vs. 49.2% traditional countries, *p* < .01) and considerably lower as to *administering* medications to manage side effects (5.4% vs. 26.5%, *p* < .001). This pattern also existed on prescribing (17.5% vs. 37.8%, *p* < .01) and administering palliative care medications (0% vs. 13.6%, *p* < .001). Finally, as to providing information to patients on breast cancer treatment and related aspects, large differences existed among all nurses and the sub-group of APN/SN across the two country groups, consistently significant at the 5% level or lower.

### Acute myocardial infarction

Compared to BC, a higher percentage of physicians treating patients with AMI in the innovator countries reported that they performed the individual tasks than in the ‘traditional skill-mix’ countries (Table 4). The differences were statistically significant in 58.6% of all tasks (17 of 29 tasks).

**Table 4:** Acute myocardial infarction: clinical tasks undertaken by percentage of physicians and nurses, by countries’ SoP, 2015/2016

	% Physicians performing tasks		% Nurses performing tasks (all nurses and APN/SN)			
	Countries with SoP expansion [1]	Countries with no/limited SoP expansion [2]	Countries with SoP expansion [1]	Countries with no/limited SoP expansion [2]	Countries with SoP expansion [1]	Countries with no/limited SoP expansion [2]
AMI	% Physicians, *N* = 30	% Physicians, *N* = 160	% All nurses, *N* = 153	% All nurses, *N* = 348	% APN/SN, *N* = 68	% APN/SN *N* = 58
*Tasks related to diagnosis/physical examination*						
Patient arrival/admission: stabilisation on arrival^[ii]^	53.3*	34.3*	33.9	31.3	27.9	41.3
Patient arrival/admission: preparing patient for heart catheterization^[ii]^	60.0**	31.2**	43.1	45.9	27.9**	53.4**
Assessment: evaluating which protocol to apply (STEMI)^[i]^	46.6	40.0	29.4***	6.3***	20.5*	6.9*
Assessment: conducting coronary angiography^[i]^	60.0***	22.5***	3.9	4.0	2.9	3.4
*Tasks related to treatment/therapy*						
Surgery: performing the catheterisation/ angioplasty^[i]^	53.3**	14.3**	2.6	3.1	2.9	1.7
Surgery: conducting percutaneous coronary intervention/angioplasty^[i]^	40.0**	13.7**	1.9	3.1	2.9	1.7
Surgery: putting in stent^[i]^	40.0**	14.3**	1.3	3.1	2.9	1.7
Surgery: coronary artery bypass graft (CABG)^[i]^	0.0	3.7	0.6	0.5	1.4	0.0
Managing complications: resuscitation^[ii]^	83.3**	57.5**	63.4	70.1	51.4	67.2
Managing complications: pericarditis^[ii]^	80.0**	46.8**	39.2	31.6	27.9	32.7
Managing complications: acute heart failure^[ii]^	83.3**	55.0**	55.5	50.5	45.5	53.4
Managing complications: arrhythmias^[ii]^	86.6**	58.1**	59.4	55.1	45.5	60.3
Managing complications: ventricular fibrillation^[ii]^	76.6*	53.7*	56.8	50.5	44.1	55.1
Managing complications: deep vein thrombosis^[ii]^	60.0	46.25	33.9	35.9	17.6*	36.2*
Managing complications: mechanical complications, e.g. septum rupture ^[i]^	70.0***	29.3***	13.7	16.6	19.1	22.4
Care post discharge (hospital-based): Managing co-morbidities/complications: vascular^[i]^	46.6	43.7	27.4***	10.0***	44.1***	28.5***
Care post discharge (hospital-based): Managing co-morbidities/complications: respiratory^[i]^	20.0*	38.7*	15.6*	8.9*	22.0*	6.9*
*Tasks related to prescribing medications (AMI)*						
Patient arrival/admission: prescribing medication^[i,ii]^	73.3*	50.6*	10.4	7.7	16.1	6.9
Patient arrival/admission: administering medication ^[i]^	40.0	21.2	54.9	63.2	35.2**	62.0**
Prescribing medication according to protocol^[i,ii]^	70.0	55.6	13.0	12.3	27.9*	10.3*
Adjusting medication based on initial effects^[i,ii]^	76.6***	56.8***	20.2**	9.7**	33.8**	12.0**
Administering medication^[i]^	43.3	29.3	54.9	61.4	41.1	56.9
Care post discharge (hospital-based): continued prescribing as per discharge letter^[i]^	26.6	45.6	13.7***	2.8***	25.0**	3.4**
Care post discharge (hospital-based): prescribing medication review^[i,ii]^	56.6	49.3	31.3***	5.7***	52.9***	5.1***
Care post discharge (hospital-based): prescribing medication change as necessary^[i,ii]^	60.0	48.7	25.4***	2.3***	44.1***	3.4***
*Tasks related to providing information to patients/coordination and follow-up*						
Handover to rehabilitation/primary care: Writing discharge letter to GP/other professional ^[ii]^	73.3**	43.1**	11.7***	2.3***	13.2**	0.0**
Handover: referral to heart rehabilitation^[i]^	36.6	31.8	50.9***	4.8***	36.7***	5.1***
Handover: plan for outpatient follow-up^[ii]^	83.3***	32.5***	30.7***	4.8***	35.2***	5.1***
Follow-up care: lifestyle support^[i]^	36.6	35.6	41.1***	15.5***	61.7***	17.2***

Source: MUNROS 2015/2016. Rationale for inclusion of tasks: ^[i]^ = suggested clinical activities as Advanced Nursing Practice: diagnosis/advanced health assessment, treatment/therapy, prescribing medications, by International Council of Nurses (ICN), ^[ii]^ = common medical tasks, reported by the majority of physicians as being part of normal duties (≥ 50% of physicians in ≥ 50% of countries). [1] England, Scotland, Netherlands, [2] Czech Republic, Germany, Italy, Norway, Poland, Turkey

*SoP* Scope-of-Practice, *APN* Advanced Practice Nurses, *SN* Specialist Nurses

***p value < .001; ** = *p* value < .01, * = *p* value < .05

Among nurses and the sub-group of APN/SN, findings across the two groups of countries were mixed. For tasks related to diagnosis, a statistically significantly higher proportion of all nurses (29.4%) and APN/SN (20.5%) in the innovator countries reported conducting assessments to evaluate which protocols to apply, compared to 6.3% and 6.9% in the traditional countries. In contrast, a higher proportion of APN/SN reported preparing patients for heart catheterization in the traditional vs. the innovator countries. For the majority of tasks related to treatment and therapy (10 of 13 tasks, 77%), differences in the proportion of nurses and APN/SN between the country groups were small and non-significant. Tasks with marked differences included managing vascular co-morbidities and complications post discharge as part of the AMI pathway, reported by 27.4% of nurses and 44.1% of APN/SN in the innovator countries compared to 10% and 28.5% in the traditional countries. The same cross-country pattern existed for managing respiratory co-morbidities.

Tasks related to prescribing medications were similar to the results for BC. APN/SN in the three countries indicated more frequently that they prescribed medications according to protocols (27.9% vs. 10.3%, *p* = .014), adjusted medications (33.8% vs. 12.0%, *p* = .004), performed continued prescribing as per discharge letter (25.0% vs. 3.4%, *p* = .001), conducted medication reviews (52.9% vs. 5.1%, *p* < .001) and prescribed medication change as necessary (44.1% vs. 3.4%, *p* < .001). Conversely, a lower proportion of APN/SN were involved in administering medications upon patient arrival (35.2% vs. 62.0% in the innovator vs. traditional countries, *p* = .003) and administering medications in general (41.1% vs. 56.9%; n.s.). For all tasks related to patient information, coordination and follow-up care, a statistically significantly higher proportion of all nurses and APN/SN in the countries with expanded SoP reported performing these activities, including writing discharge letters, referrals, plans for follow-up care and lifestyle support.

## Discussion

The nine countries in this study show large variations in health professionals’ perceived changes to staff roles, independence in new roles and the tasks performed in hospitals in the care for patients with BC and AMI. Health professionals from the innovator countries (Netherlands, England and Scotland) with major SoP reforms implemented between 2010 and 2015, more frequently considered staff role changes had occurred and that these had higher levels of independence in executing tasks formerly done by physicians compared to health professionals from the six countries with no reforms. In terms of individual tasks, nurses and in particular APN/SN working on BC from the three countries reported that they performed more tasks and more frequently tasks that were a priori defined as medical tasks compared to those in the six countries. These differences across the two groups of countries exist for all professional nurses and the sub-group of APN/SN, demonstrating that the extent of task shifting is consistently higher in countries with expanded SoP. For AMI, comparable patterns exist between the two groups of countries on prescribing medications, patient information and follow-up care. On diagnosis and treatment-related tasks, however, nurses and APN/SN show a similar involvement across the two country groups.

Perceived changes to staff roles may have been triggered by policy reforms implemented between 2010 and 15 that expanded SoP, but may also have been influenced by other factors, including changes at the organisational levels or condition-specific factors. Hence, the study’s findings show associations, but was not designed to demonstrate causality. Yet, the findings are consistently reported from health professionals working on two different conditions, suggesting that changes to roles are independent from condition-specific factors. The consistently higher proportion of nurses reporting that they prescribed medications in the three countries, including APN/SN, suggests that the uptake of the related policy reforms occurred in hospital-based practice. However, the roles and underlying mechanisms for these cross-country differences should be analysed in future research, e.g. at the policy, organisational and team levels.

The findings of this study are largely in line with previous research. Our findings confirm and expand previous international studies on large cross-country differences in the official practice profiles of nurses and APN/NP [13–15]. Similarities exist for the Netherlands, England and Scotland insofar as official practice was expanded as per SoP, yet with variations in their uptake, particularly on nurse prescribing of medicines. In our study’s sample of APN/SN between 10.8% and 15.2% (for BC patients) and 16.1% and 27.9% (AMI) of APN/SN reported to newly prescribe medications. Percentages were higher for *revising* medications or *adjusting* medications. In a 2013 study on nurse prescribing in Dutch hospitals conducted 1 year after implementation of the 2012 law, more nurse specialists reported to prescribe a limited set of ‘standard’ medications of low risk while fewer nurse specialists wrote prescriptions for a wider range of medicines. The study found large variations if nurse specialists prescribed medicines (at all) and the extent of prescribing in daily practice, ranging from prescriptions for up to 16 patients a day by one nurse specialist to limited prescribing once a week. Several nurse specialists reported not to prescribe medicines at all as they had not been granted the approval by their respective hospital boards [23]. Although nurse specialists are by law authorised to prescribe independently, the 2013 study showed that in practice prescribing of medicines is often performed under close oversight measures, depending on local contextual factors, e.g. prior hospital board approval and/or in close collaboration and oversight by physicians. Moreover, the implementation of the law in 2012 was relatively recent which may also explain why in our study, a moderate percentage of health professionals (51.3% and 38.2% of health professionals working on BC and AMI) from the Netherlands reported that new professional roles are working independently without physician supervision. In England and Scotland, prescribing of medicines has a longer history dating back to 2006, of which the 2012 law expanded prescribing rights to independent prescribers, hence may explain the higher percentage of at least 50% health professionals reporting independence in executing new tasks that were formerly provided by physicians, consistently for both conditions.

Our findings suggest that task shifting and changes to professional boundaries between physicians and nurses, and in particular APN/SN and physicians, is practiced in the Netherlands, England and Scotland to a greater extent than in the other six countries. However, most tasks were reported to be performed by both professions rather than executed by one profession only, suggesting that partial instead of full task shifting occurred. Yet, the underlying reasons and mechanisms are unclear. It may reflect division of work between more complex and routine cases, it may also reflect multidisciplinary team work. A more detailed analysis of tasks by level of specialisation, risk and complexity of patients' conditions; and whether the tasks are performed by one professional or jointly within teams, would be a next step to better understand which tasks for what patient groups are more commonly performed by which professional cadre. The study by McDonnel that found NP in England performing tasks that were done by junior doctors in the past, suggests that routine instead of highly specialised, complex medical activities may have been shifted to the nursing profession [24]. Qualitative studies could add more in-depth insights into the contextual factors of why tasks are performed by whom, the division of work, nature of team work and the possibly influencing factors.

In the six countries with no changes to SoP, for most tasks surveyed, a statistically significantly lower proportion of nurses and APN/SN reported that they performed these tasks. Yet, exceptions existed. For instance, very low proportions of APN/SN and a small proportion of nurses reported to prescribe medicines which are not legally allowed in these countries. Reasons are unclear but the results may reflect informal prescribing, as has been reported in some countries, such as the Netherlands or Spain, which was one of the factors triggering reforms to implement laws officially authorising nurse prescribing in the Netherlands [13, 32, 33]. On the other hand, in Germany for instance, nurses may be allowed to give medicines to patients from a defined list if pre-authorised in writing by the physician-in-charge (so-called ‘needs-based medications’) [34], which some nurses may have falsely interpreted as prescribing medicines.

The study faces several limitations. First, the cross-sectional design of the study limits attribution of causality between perceived changes to staff roles at hospital levels and implementation of policy reforms. Yet, health professionals completed the surveys in 2015 and 2016; hence, the time period covered was 2010/2011 to 2015/2016, largely in line with the 2010–2015-year period for which reforms were covered. Second, the study’s non-representative sample and in particular the small number of physicians working on AMI limits the generalisability of the findings. The large and statistically significant differences in the AMI tasks between physicians from the two country groups may have been biased, for instance if different medical cardiology specialisations participated. Third, the comparability of the individual tasks between the conditions was not fully possible. The  tasks identified on breast cancer missed out referrals and writing discharge letters which were subsumed under “administrative tasks”, among others, whereas only few tasks existed for AMI and diagnosis. Finally, the information is based on health professionals’ self-reports which were not cross-checked, hence may risk recall bias. Nevertheless, the study is the first of its kind to quantify nurses’ and APN/SN self-reported involvement in a detailed set of ‘medical’ tasks for patients with two conditions from a cross-country perspective.

## Conclusion

Nurses and APN/SN in the Netherlands, England and Scotland are performing a wide range of tasks at the interface to the medical professions, suggesting that task shifting has occurred, for instance on chemotherapy or prescribing medicines. For most tasks, however, a partial instead of full task shifting is practice.

## Abbreviations

AMI: Acute myocardial infarction
APN/NP: Advanced Practice Nurse(s)/Nurse Practitioner(s)
BC: Breast cancer
n.s.: (Statistically) non-significant
SN: Specialist Nurse(s)
SoP: Scope-of-Practice
UK: United Kingdom
USA: United States of America

## Footnotes

1 See website for further details: https://www.abdn.ac.uk/munros

2 Official term used: *Verpleegkundig Specialist*

3 Official term used: Advanced Nurse Practitioner
